# Supplementary material for: Oxytocin pathway gene networks in the human brain
Source: Nat Commun. 2019 Feb 8;10:668. doi: 10.1038/s41467-019-08503-8 (PMC6368605; doi:10.1038/s41467-019-08503-8)
Supplement: Supplementary file 1 — Supplementary Information [file 41467_2019_8503_MOESM1_ESM.pdf]

# Oxytocin pathway gene networks in the human brain

Daniel S. Quintana et al.

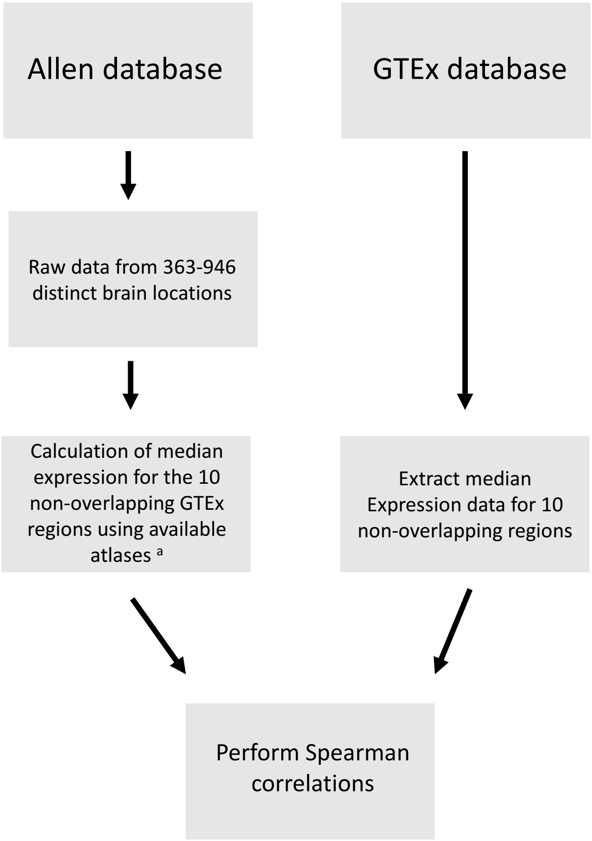


**Supplementary Figure 1. The comparison of Allen and GTEx datasets.** Median expression data from 10 distinct regions from the GTEx database were compared to the median expression of the same regions from the Allen database. Medians from the Allen dataset were extracted from the raw data by calculating the median expression of the GTEx regions using available atlases. Spearman correlations were then calculated to assess the relationship of these datasets. [a] AAL atlas: Amygdala, Caudate, Cerebellum, Hippocampus, Putamen; Talairach atlas: BA24, BA9, Hypothalamus, Substantia Nigra; “Hammers_mith atlas”: Nucleus Accumbens

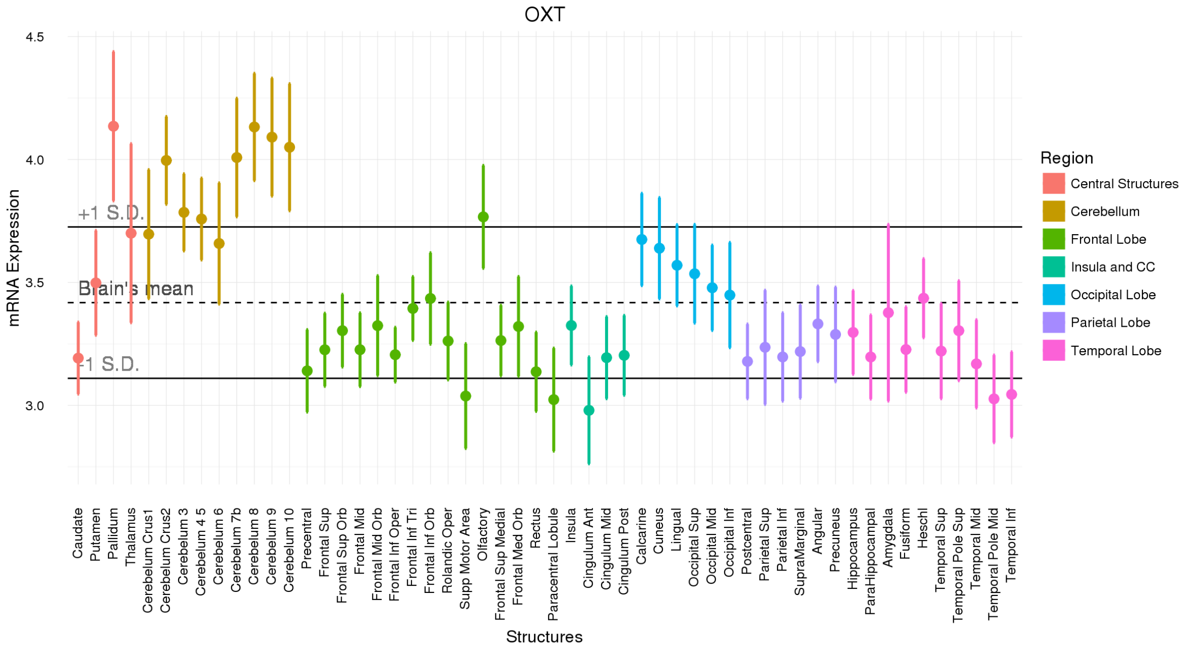


**Supplementary Figure 2. The central expression of OXT.** Means and standard errors are presented.

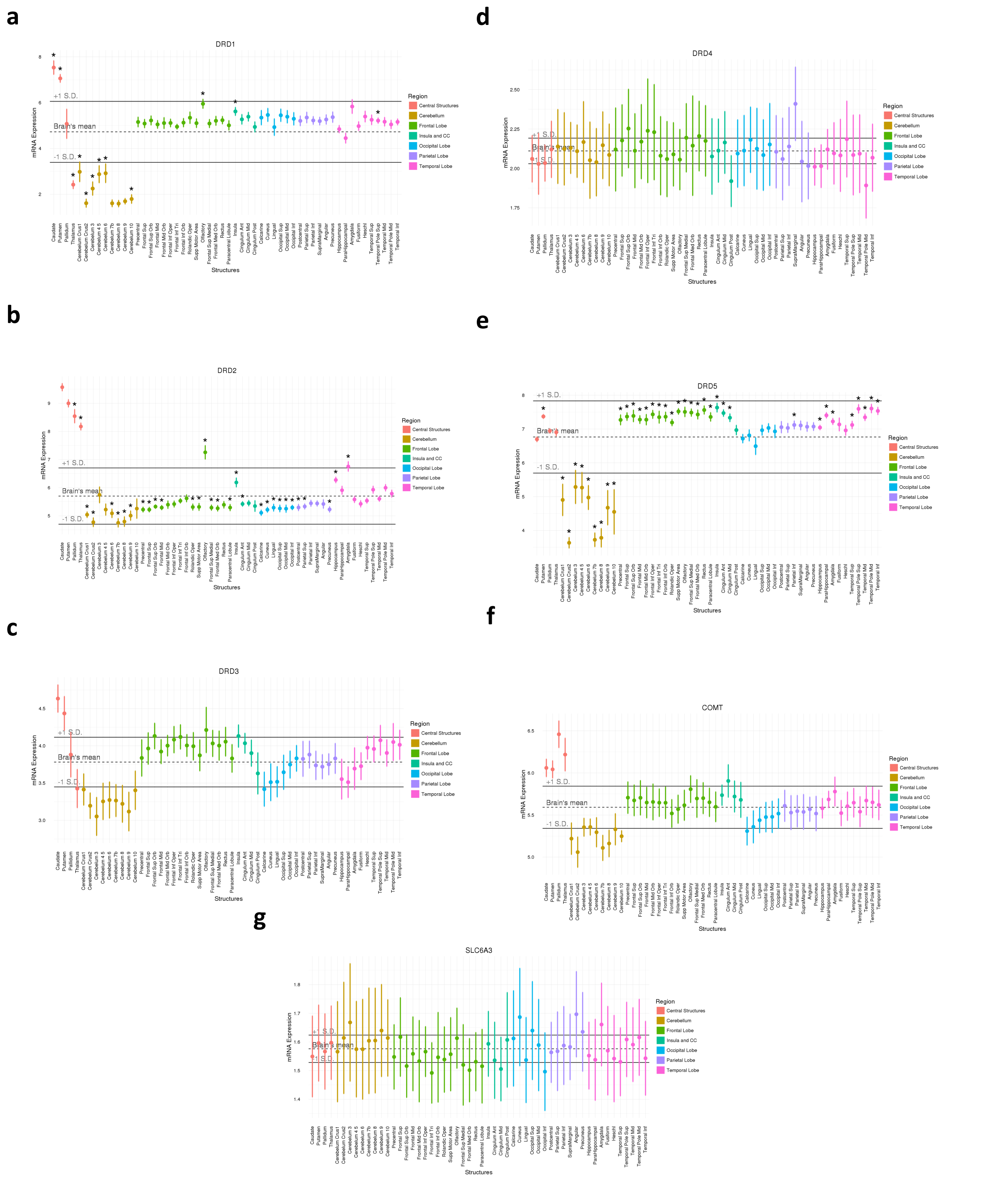


**Supplementary Figure 3. The central expression of dopaminergic genes DRD1 (a), DRD2 (b), DRD3 (c), DRD4 (d), DRD5 (e), COMT (f), and DAT1/SLC6A3 (g)** Means and standard errors are presented. Statistically significant two-tailed one-sample *t*-tests are shown. * $p > 0.05$ (FDR corrected).

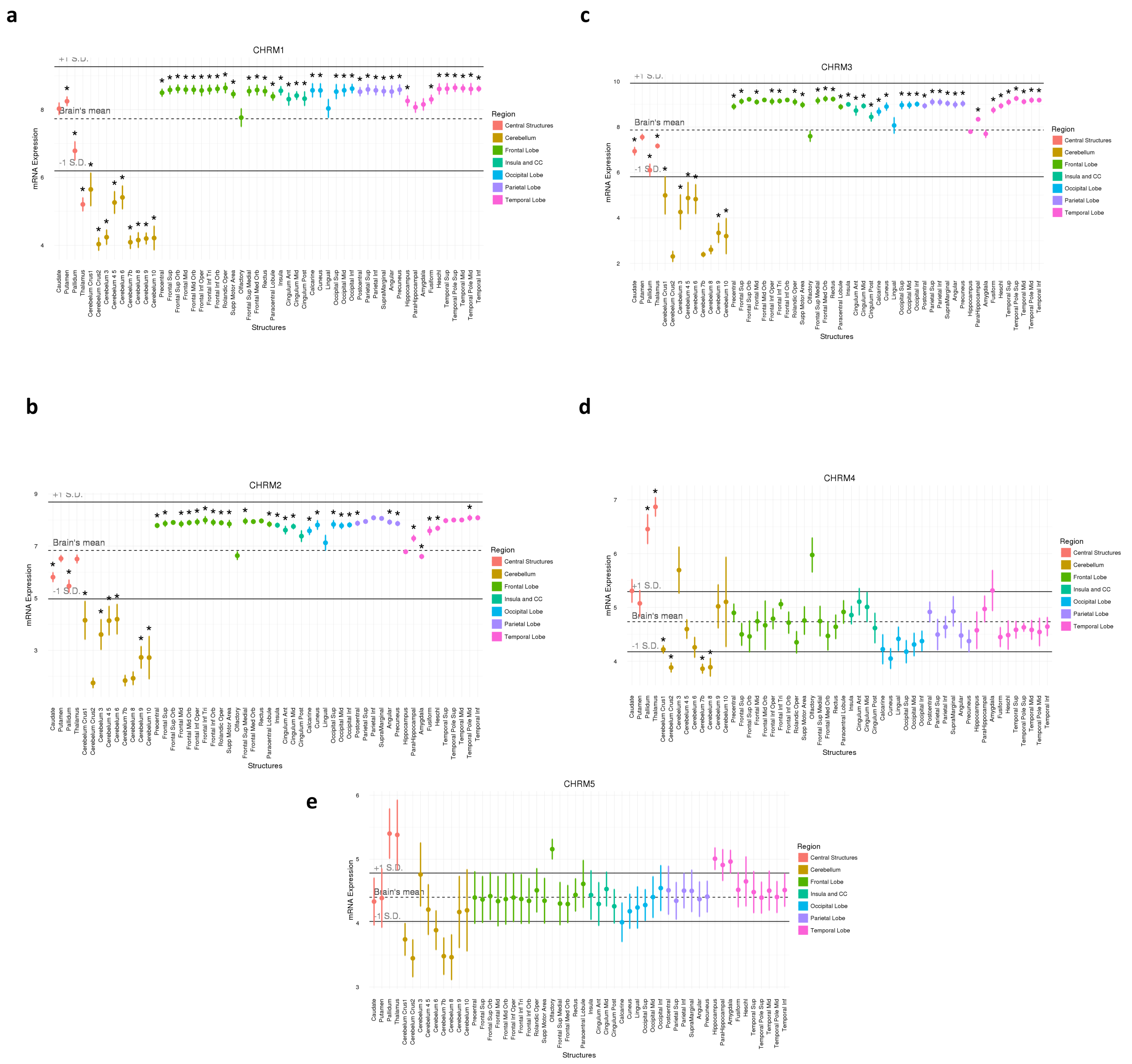


**Supplementary Figure 4. The central expression of muscarinic acetylcholine pathway genes CHRM1 (a), CHRM2 (b), CHRM3 (c), CHRM4 (d), and CHRM5 (e).** Means and standard errors are presented. Statistically significant two-tailed one-sample *t*-tests are shown. * $p > 0.05$ (FDR corrected).

a

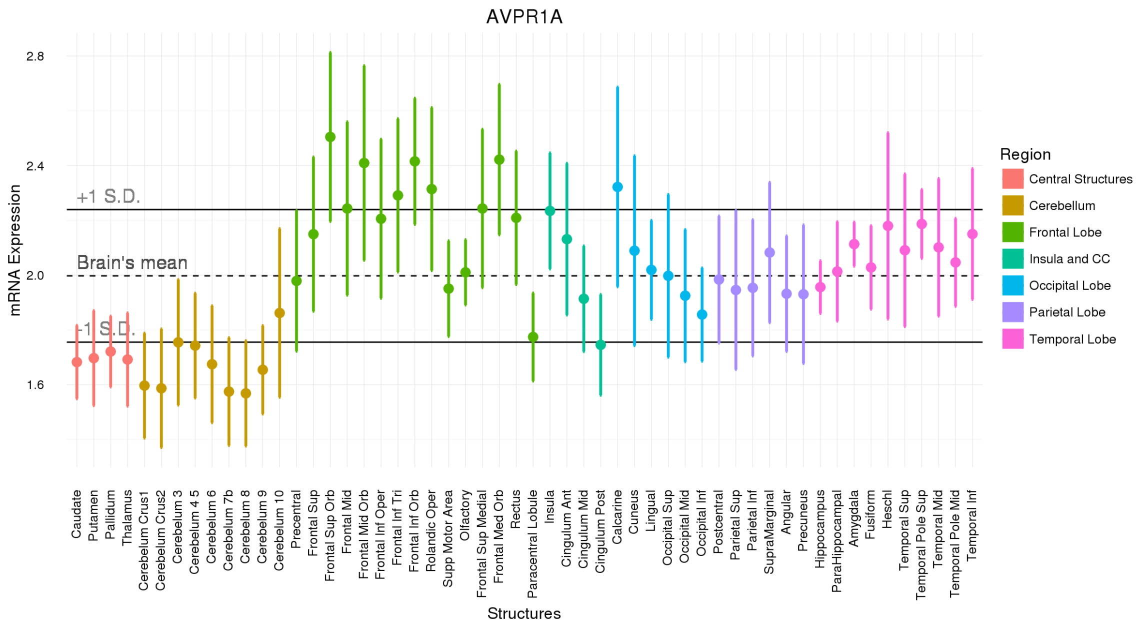


b

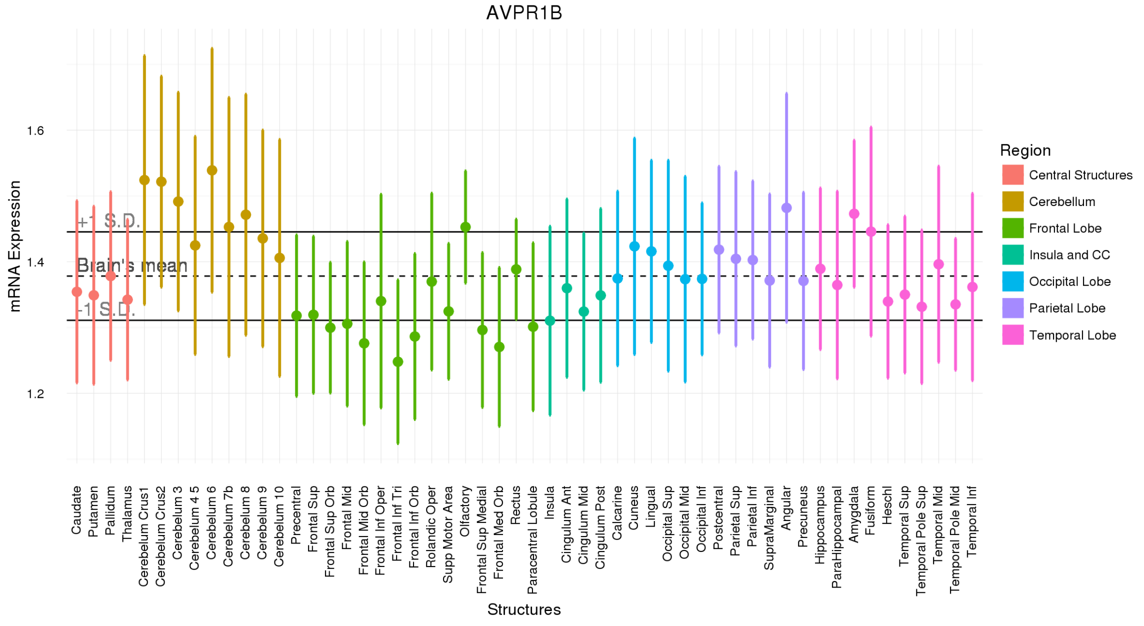


**Supplementary Figure 5. The central expression of vasopressin genes AVPR1A (a), and AVPR1B (b).** Means and standard errors are presented.

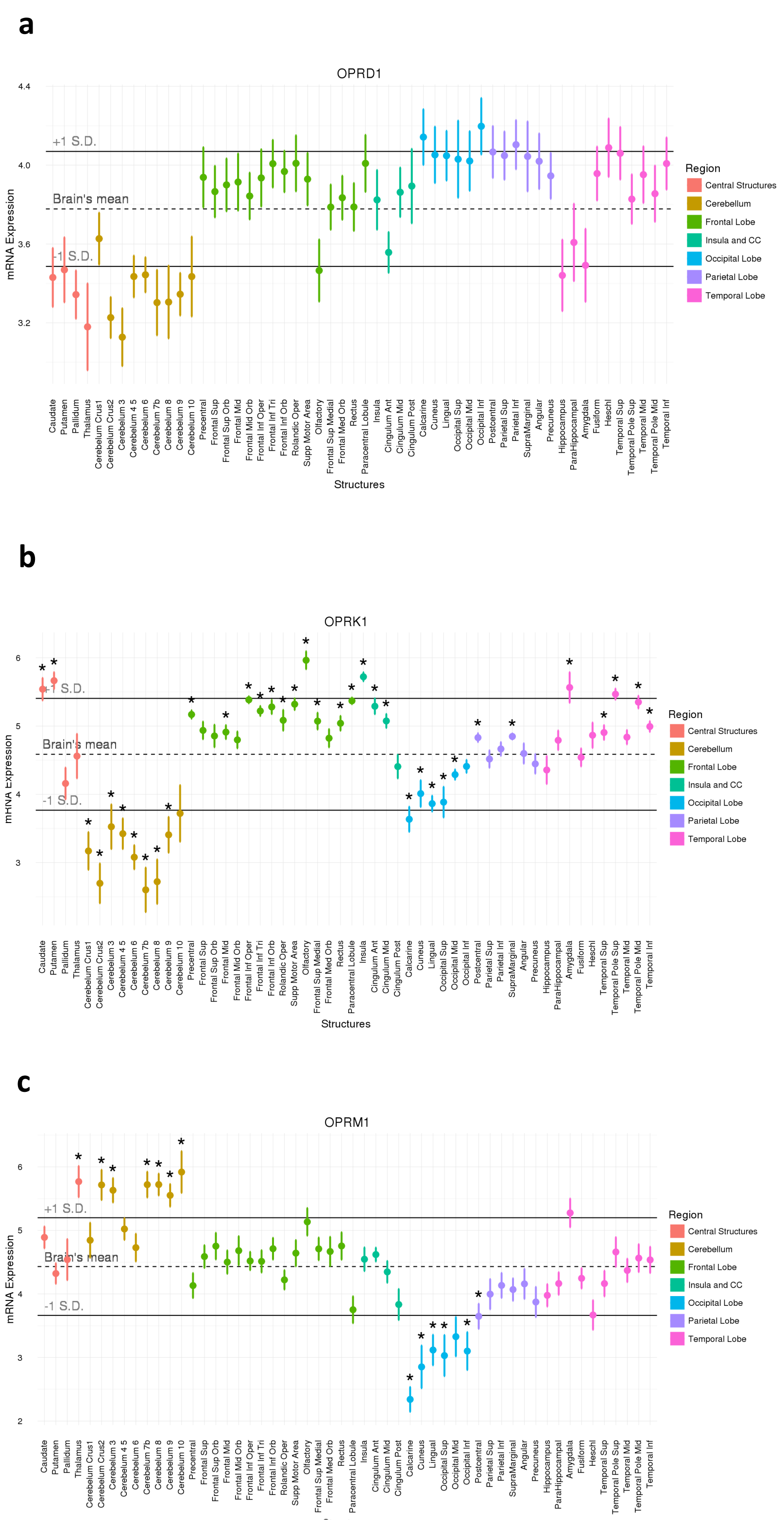


**Supplementary Figure 6. The central expression of opioid genes OPRD1 (a), OPRK1 (b), and OPRM1 (c).** Means and standard errors are presented. * > 0.05 (FDR corrected).

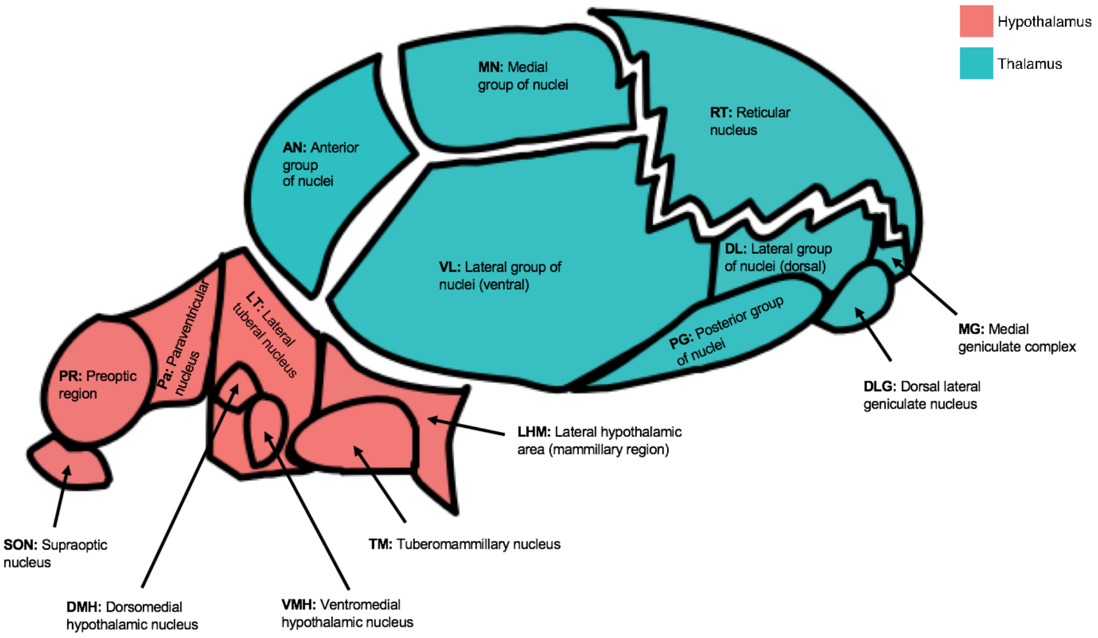


**Supplementary Figure 7. Anatomical legend for thalamic and hypothalamic substructures.**

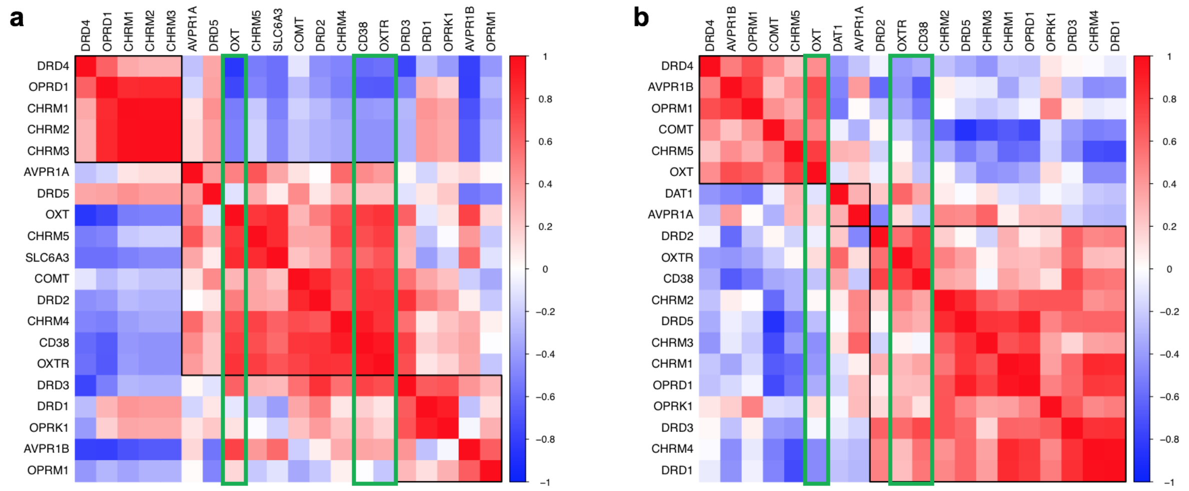


**Supplementary Figure 8. Independent sample validation of the central co-expression of oxytocin, dopaminergic, muscarinic acetylcholine, vasopressin, and opioid gene sets.** As described in Figure S2, median expression data was extracted from the Allen (a) and GTEx (b) datasets. Values represent Spearman correlation coefficients. Complete linkage clustering was used to identify 3 clustering groups (black squares). The key constituents of the oxytocin pathway (*OXTR, CD38, OXT*) where highlighted by green rectangles. In both datasets, *OXTR* and *CD38* clustered together, along with DRD2, and CHRM4 genes.

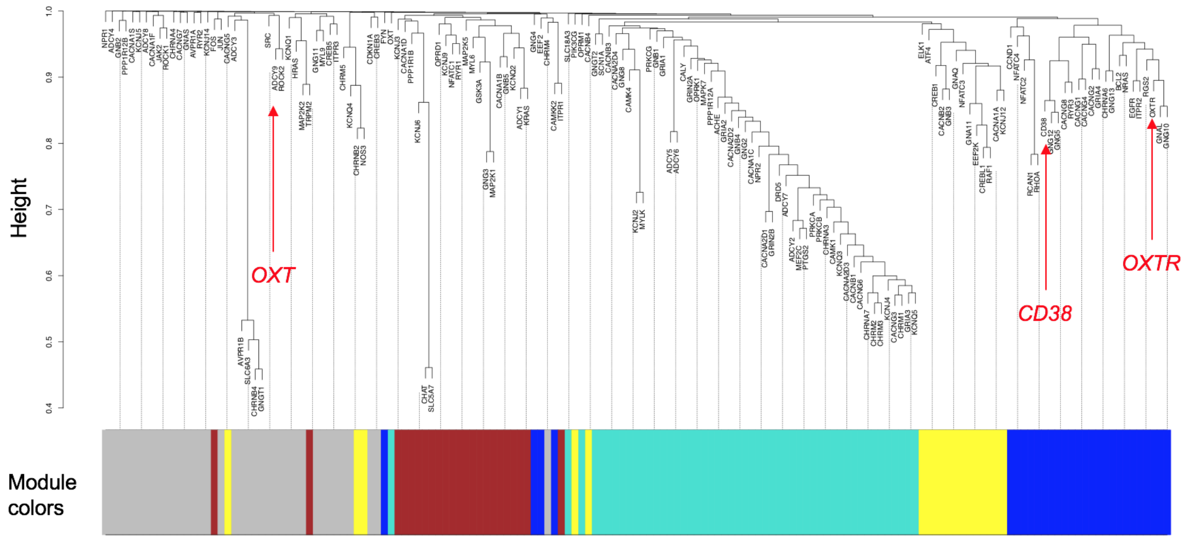


**Supplementary Figure 9. WGNCA cluster dendrogram with modules and labelled genes.** mRNA expression values of all oxytocin pathway genes in the brain were submitted for weighted gene co-expression network analysis. *OXTR* and *CD38* were clustered together in the same module. *OXTR*, *CD38*, and *OXT* are highlighted with red arrows and labelled in red

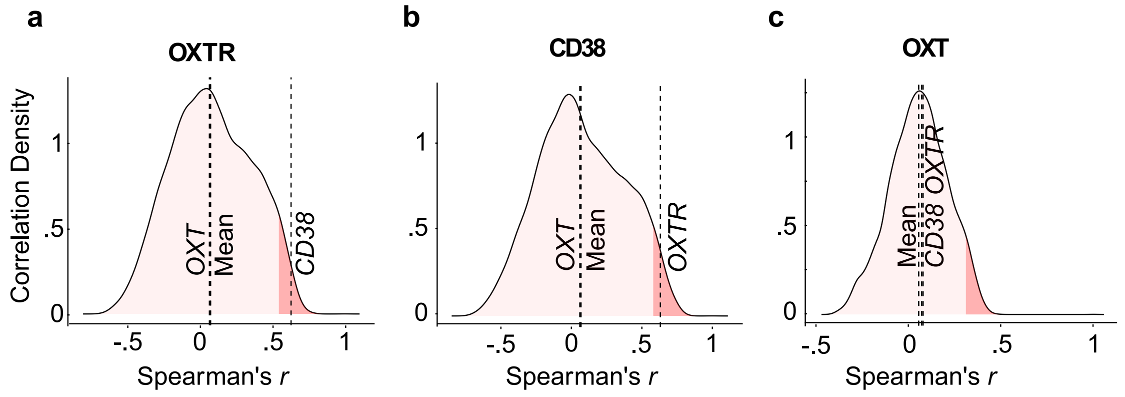


**Supplementary Figure 10. Co-expression of oxytocin pathway genes against 20737 protein coding genes.** Density plots visualize the distributions of correlations between *OXTR* (a), *CD38* (b), and *OXT* (c), and all other mRNA probes. For each oxytocin pathway gene of interest, the position of the other two oxytocin pathway genes of interest are shown. The correlation between OXTR and CD38 is in the top 5% of all correlations, which is indicated by the dark red portions of the distributions.

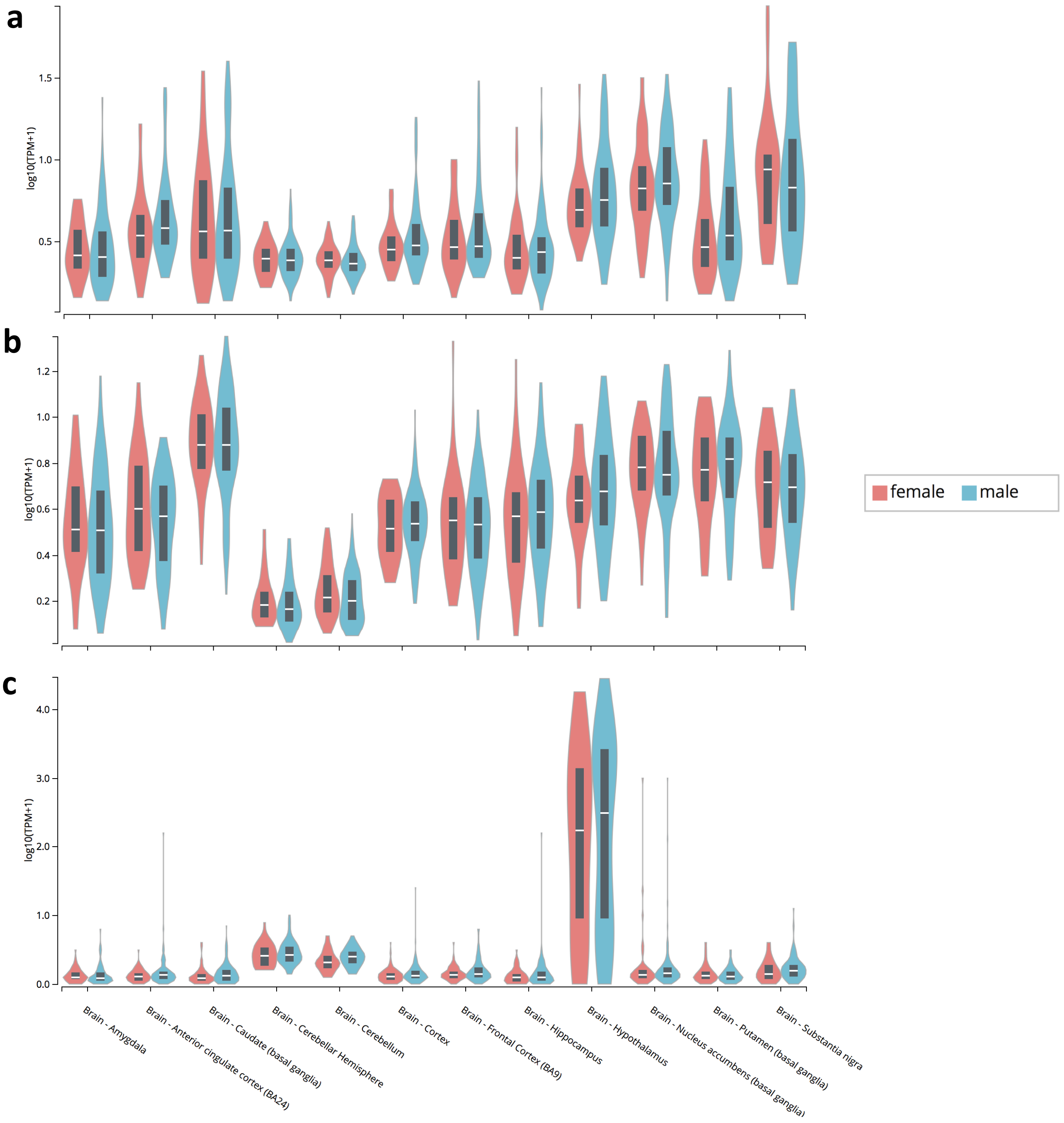


**Supplementary Figure 11. Sex differences in central oxytocin pathway mRNA expression.** Box plots demonstrate median central expression of *OXTR* (a), *CD38* (b), and *OXT* (c) in males (blue boxes) and females (red boxes) over 12 brain regions, with 25th and 75th percentiles. Data was extracted from the GTEx database (https://gtexportal.org), with gene expression presented as Transcripts per million (TPM) as log transformed units.

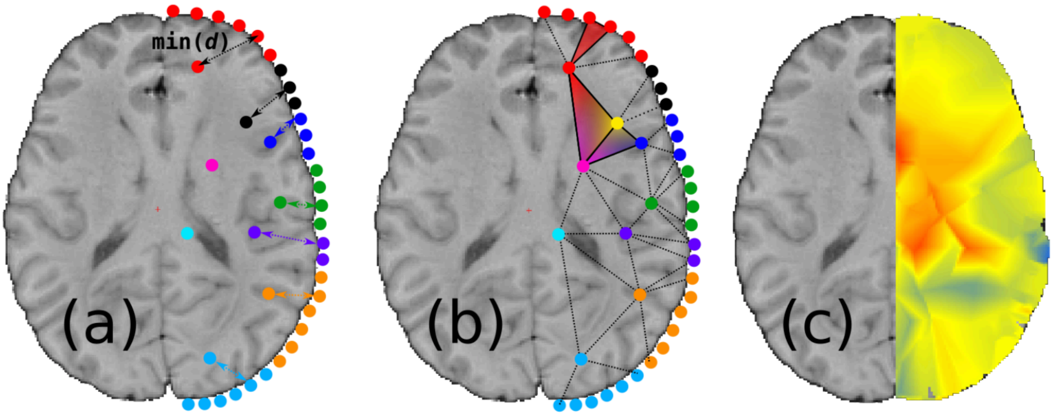


**Supplementary Figure 12. Creation of expression maps.** First, brain boundaries were labelled (a). Spaces were then divided into simplices, which were triangles in two-dimensional cases (note that not all possible triangles are marked in the image) (b). We then performed linear interpolation within each simplex (c).

**Supplementary Table 1. Probe details for top Spearman's correlations between oxytocin pathway genes and all remaining genes**

| | OXTR | | | CD38 | | | OXT | | |
|---|---|---|---|---|---|---|---|---|---|
| | Gene | Probe ID | $r_s$ | Gene | Probe ID | $r_s$ | Gene | Probe | $r_s$ |
| Top 10 positive | LUPZ2 | 1029580 | 0.793 | NTSR2 | 1045487 | 0.821 | EPS8L2 | 1039716 | 0.492 |
| | NTSR2 | 1045487 | 0.779 | SLC14A1 | 1020063 | 0.811 | FLYWCH2 | 1011181 | 0.473 |
| | GLUD2 | 1056377 | 0.776 | C12orf39 | 1038445 | 0.807 | IL28RA | 1033899 | 0.466 |
| | GLUD1 | 1056379 | 0.771 | AQP4 | 1024273 | 0.802 | N4BP2 | 1041644 | 0.465 |
| | MLEC | 1048178 | 0.765 | LIX1 | 1033799 | 0.799 | MAP2K3 | 1052652 | 0.461 |
| | THBS4 | 1050797 | 0.763 | PSAT1 | 1027224 | 0.794 | CCR10 | 1056267 | 0.453 |
| | KIAA1407 | 1040552 | 0.759 | PRTFDC1 | 1041054 | 0.782 | CD14 | 1022645 | 0.451 |
| | ZC3HAV1 | 1041166 | 0.748 | HSDL2 | 1037724 | 0.78 | C5orf56 | 1025314 | 0.446 |
| | HEYL | 1044845 | 0.743 | ITGB8 | 1054914 | 0.78 | CLDN3 | 1057991 | 0.443 |
| | NAV2 | 1020830 | 0.741 | GLUD1 | 1056379 | 0.777 | CBX5 | 1010672 | 0.442 |
| Top 10 negative | GABRD | 1056608 | -0.67 | KCNJ3 | 1054823 | -0.738 | LHX2 | 1048690 | -0.414 |
| | KCNJ3 | 1054823 | -0.66 | AC021534.2 | 1019865 | -0.724 | NEK2 | 1053760 | -0.412 |
| | CHRD | 1049481 | -0.652 | CHRD | 1049481 | -0.706 | KCNV1 | 1044726 | -0.41 |
| | AC021534.2 | 1019865 | -0.647 | PAK7 | 1026139 | -0.705 | LOC339524 | 1020418 | -0.401 |
| | KIAA0408 | 1025969 | -0.647 | GABRD | 1056608 | -0.7 | AK5 | 1027247 | -0.397 |
| | PAK7 | 1026139 | -0.643 | IGSF21 | 1037180 | -0.7 | CHSY3 | 1022072 | -0.394 |
| | KIAA0802 | 1019330 | -0.632 | GALNT9 | 1018522 | -0.695 | TRIM48 | 1016645 | -0.394 |
| | SRPK1 | 1051136 | -0.632 | ACTL6B | 1043413 | -0.692 | ENC1 | 1049632 | -0.393 |
| | KIAA1456 | 1015411 | -0.631 | NEUROD1 | 1053753 | -0.691 | KCNJ4 | 1054822 | -0.392 |
| | SLC6A7 | 1051449 | -0.631 | MICAL2 | 1048322 | -0.696 | CTNNA2 | 1057852 | -0.391 |

**Supplementary Table 2. Top 5 mental state correlations for *OXTR*, *CD38*, and *OXT* brain mRNA maps submitted to NeuroSynth with rank and percentage out of all 20,737 protein coding genes**

| Cognitive state | Number of fMRI studies | *OXTR* | | | | *CD38* | | | | *OXT* | | | |
|---|---|---|---|---|---|---|---|---|---|---|---|---|---|
| | | $r_s$ | *p*-value | Rank | Rank percentage | $r_s$ | *p*-value | Rank | Rank percentage | $r_s$ | *p*-value | Rank | Rank percentage |
| Anticipation | 301 | 0.215 | < .0001 | 549 | 2.65 | 0.283 | < .0001 | 13 | 0.06 | 0.082 | 0.0597 | 6382 | 30.78 |
| Anxiety | 449 | 0.297 | < .0001 | 60 | 0.29 | 0.238 | < .0001 | 495 | 2.39 | 0.022 | 0.3375 | 8056 | 38.85 |
| Emotional | 1708 | 0.293 | < .0001 | 178 | 0.86 | 0.224 | < .0001 | 793 | 3.82 | 0.005 | 0.4627 | 7802 | 37.62 |
| Facial expressions | 250 | 0.070 | 0.0906 | 2633 | 12.70 | 0.040 | 0.2261 | 5537 | 26.70 | 0.065 | 0.1098 | 3090 | 14.90 |
| Fear | 363 | 0.205 | < .0001 | 481 | 2.32 | 0.117 | 0.0128 | 2673 | 12.89 | 0.079 | 0.0676 | 4421 | 21.32 |
| Incentive | 148 | 0.242 | < .0001 | 49 | 0.24 | 0.239 | < .0001 | 64 | 0.31 | 0.039 | 0.2303 | 7712 | 37.19 |
| Learning | 1144 | 0.074 | 0.0809 | 7825 | 37.73 | 0.110 | 0.0182 | 5773 | 27.84 | 0.161 | 0.0011 | 3328 | 16.05 |
| Monetary | 300 | 0.206 | < .0001 | 452 | 2.18 | 0.263 | < .0001 | 38 | 0.18 | 0.055 | 0.1493 | 6106 | 29.44 |
| Motivation | 189 | 0.234 | < .0001 | 34 | 0.16 | 0.224 | < .0001 | 69 | 0.33 | 0.047 | 0.1881 | 6368 | 30.71 |
| Reward | 922 | 0.324 | < .0001 | 240 | 1.16 | 0.347 | < .0001 | 114 | 0.55 | 0.078 | 0.0699 | 6102 | 29.43 |
| Seeking | 99 | 0.119 | 0.0115 | 1746 | 8.42 | 0.168 | 0.0007 | 234 | 1.13 | 0.088 | 0.0480 | 3853 | 18.58 |
| Sexual | 81 | 0.245 | < .0001 | 19 | 0.09 | 0.177 | 0.0004 | 942 | 4.54 | 0.036 | 0.2448 | 7933 | 38.26 |
| Stress | 321 | 0.266 | < .0001 | 358 | 1.73 | 0.263 | < .0001 | 396 | 1.91 | 0.020 | 0.3490 | 8116 | 39.14 |
| Taste | 80 | 0.224 | < .0001 | 393 | 1.90 | 0.238 | < .0001 | 229 | 1.10 | 0.097 | 0.0321 | 4397 | 21.20 |
| Unpleasant | 132 | 0.156 | 0.0014 | 919 | 4.43 | 0.128 | 0.0072 | 1858 | 8.96 | 0.103 | 0.0252 | 3054 | 14.73 |

*Note.* After retrieving the mental state terms with the 5 strongest relationships with the OXTR map, we skipped duplicate terms when retrieving the 5 strongest relationships with the CD38 map and the repeated this process (skipping duplicates) with the OXT map. Ranks and rank percentages represent the rank for correlations between the *OXTR, CD38,* and *OXT* gene maps and the given terms compared to all 20737 genes maps.

**Supplementary Table 3. Detailed donor profiles**

| ID | Ethnicity | Gender | Age | Post-mortem interval (hours) | Number of brain samples | Hemisphere |
|---|---|---|---|---|---|---|
| 1 | Caucasian | Male | 57 | 25.5 | 363 | Left |
| 2 | Caucasian | Male | 31 | 17.5 | 529 | Left |
| 3 | Hispanic | Female | 49 | 30.0 | 470 | Left |
| 4 | Caucasian | Male | 55 | 18.0 | 501 | Left |
| 5 | African American | Male | 24 | 25.0 | 946 | Both |
| 6 | African American | Male | 39 | 18.0 | 893 | Both |

**Supplementary Table 4. Fit indices for scale-free topology**

| Power | Soft-thresholding power (adjusted $R^2$) | Mean connectivity | Median connectivity | Maximum connectivity |
|---|---|---|---|---|
| 1 | 0.665 | 51.4 | 52.3 | 72.6 |
| 2 | 0.145 | 24.3 | 23.4 | 44.5 |
| 3 | 0.185 | 13.7 | 11.9 | 31.3 |
| 4 | 0.694 | 8.61 | 6.58 | 24.3 |
| 5 | 0.103 | 5.82 | 4.19 | 19.8 |
| 6 | 0.793 | 4.16 | 2.71 | 16.7 |
| 7 | 0.741 | 3.11 | 1.92 | 14.4 |
| 8 | 0.804 | 2.4 | 1.37 | 12.7 |
| 9 | 0.0672 | 1.91 | 0.906 | 11.2 |
| 10 | 0.828 | 1.55 | 0.608 | 10.1 |
| 12 | 0.781 | 1.08 | 0.29 | 8.27 |
| 14 | 0.795 | 0.791 | 0.161 | 6.93 |
| 16 | 0.213 | 0.604 | 0.0899 | 5.9 |
| 18 | 0.212 | 0.475 | 0.0533 | 5.08 |
| 20 | 0.93 | 0.382 | 0.0306 | 4.42 |
