# Supplementary material for: Oxytocin pathway gene networks in the human brain
Source: Nat Commun. 2019 Feb 8;10:668. doi: 10.1038/s41467-019-08503-8 (PMC6368605; doi:10.1038/s41467-019-08503-8)
Supplement: Supplementary file 2 — Description of Additional Supplementary Files [file 41467_2019_8503_MOESM2_ESM.pdf]

Description of Additional Supplementary Files

File Name: Supplementary Data 1
Description: Sheets are described individually below

1. keg_gene_sets: A list of the genes included in the oxytocinergic, dopaminergic, and cholinergic pathway genesets, derived from https://www.kegg.jp 2.
2. AAL_atlas_gene_exp: Gene expression values for AAL atlas brain regions from each donor for the 20 genes of interest
3. AAL_atlas_means: mRNA expression values (mean and standard deviation) for the 20 genes of interest in AAL atlas brain regions along with t statistics from the one-sample t-tests, p-values, FDR corrected p-values, and Cohen's d values. 3. thalamic_hypothalamic_gene_exp: mRNA expression values (mean and standard deviation), FDR adjusted p-value and z scores for OXTR, CD38, and OXT in thalamic and hypothalamic structures
4. WGCNA_modules: List of modules and genes derived from weighted gene co-expression network analysis.
5. social_vs_nonsocial: List of mental states categorized as social or non-social, along with OXTR, CD38, and OXT mRNA expression in brain regions associated with these mental states.

File Name: Supplementary Data 2
Description: The correlation between oxytocin pathway gene expression in the brain (OXTR, CD38, and OXT and all other protein coding genes (n = 20736). Data is sorted largest to smallest correlation (Spearman's r) with associated p-values also shown
Sheets are described individually below

1. OXTR: Correlations with OXTR expression
2. CD38: Correlations with CD38 expression
3. OXT: Correlations with OXT expression
